# Some Diseases of the Eye in Lower Animals

**Published:** 1883-07

**Authors:** William Oliver Moore

**Affiliations:** Prof. Comparative Ophthalmology, Columbia Veterinary College, N. Y., Associate Prof. of Ophthalmology and Otology, New York Post Graduate Medical School, etc.


### ART. XIX.—SOME DISEASES OF THE EYE IN LOWER ANIMALS.

BY WILLIAM OLIVER MOORE, M.D.,
*Prof. Comparative Ophthalmology, Columbia Veterinary College, N. Y.,*
*Associate Prof. of Ophthamology and Otology, New York Post Graduate Medical School, etc.*

IN the January number of this JOURNAL (Vol. IV., No. I) we spoke of the inflammations of the conjunctiva; in this article we propose to mention some of the consequences.

*Granular Lids—Trachoma.*—This is a condition of the lining membranes of the eyelids, caused by a chronic inflammation of the conjunctiva, usually preceded by either catarrhæ or purulent conjunctivitis. The condition is not easily seen without first having examined the inner surface of the lids.

On everting the upper lid, the naturally smooth lining will be found to be rough and granular, often the granules being quite large and distinct, in some cases the lid surface presents the appearance of frog spawn. The upper lid is the one most commonly affected, though it may occur in both. This roughness of the lid causes a dullness and ulceration of the cornea, in many cases the cornea becomes very vascular and this condition is called pannus. Many animals from the western prairies have this disease, caused by the sand being blown into their eyes, thus producing an inflammation of the conjunctiva. In the human subject the disease is very common and gives rise to much distress and diminution of vision. The treatment consists in touching the panular surface. This is best done by everting the upper lid and applying to the inflamed surface sulphate of copper in crystal, gently rubbing it over the lid. If the lower lid is affected the same may be done or, instead of the copper a "mitigated stick," composed of nitrate of silver, one part, and nitrate of potash, two parts, will be found useful. These applications should be made twice a week at least, and will have to be kept up for a long time. Besides these measures the lids should be bathed with cold water and the secretions carefully wiped away. If there should be great spasm of the eyelids causing pressure on the globe an operation can be done on the outer canthus of the eye, in other words a division of the orbicular muscle which surrounds the lids can be made. The operation is called canthoplasty, and is of great service in some cases. Solutions of nitrate of silver, five and ten grains to the ounce may be used. Sometimes a solution of tannin and glycerine serves a good purpose (Ac. Tannic gr. x—Glycerine ℥i). In fact we have to change from one to the other, as the disease is so protracted. We are now using in the human subject a new remedy, namely, an infusion made from a Brazilian bean called the licorice bean. This infusion is dropped into the eye and produces a new inflammation of a membranous character, which, when subsiding, causes a removal of the granulations and a decrease in the vascularity of the cornea. It is just on its trial in this country, but so far as I have used it I have had excellent results and I look upon it as a great gain to our knowledge. This disease is not so common in animals as in man.

*Keratitis—Ulcers of the Cornea, etc.*—Keratitis is an inflammation of the cornea, and may be of the superficial or deep parts of its structure. Whenever we have an abrasion of the corneal surface we speak of it as an ulcer. These ulcers may occur from injuries, or may be the direct result of any severe inflammation of the eyelids. During the progress of purulent conjunctivitis, or "blinds," in cattle, we have the cornea very seriously involved, ulcers forming deep in the tissue and oftentimes perforating its substance, causing thereby a loss of the aqueous humor and a prolapse of the iris into the wound. This adhesion of the iris to the cornea brought about in this way is called anterior synechia, and the opacity formed on the healing of the ulcer a leucoma adhærens. All inflammations of the deep structures of the cornea, leave behind them opacities, which show more or less distinct according to the extent of the tissue change. Sometimes the whole corneal surface is milky, or there may be only one prominent white spot, the seat of some former ulcer. A great deal of confusion seems to exist in the minds of many in regard to these opacities, the result of keratitis. By many they are called cataract. No greater mistake could be made, and if a careful examination is made none need make such an error. These opacities are always on the corneal surface and may be at any point, whereas cataract, is behind the cornea, is an opacity in the crystalline lens, and is *always* the size of the pupillary opening.

When the keratitis is associated with the formation of pus in the anterior chamber of the eye it is termed keratitis hypopion; it is a violent inflammation, and needs special care. Keratitis occurs in broken down and cachectic animals, in the human subject it often occurs in children as the result of inherited syphilis. This latter form is obstinate and causes, much anxiety to the physician. Keratitis and ulcers of whatever form, are treated locally, by the instillation of a solution, of sulphate of atropia (gr. ii, ℥i aq.) three times a day. This not only relieves pain, but also dilates the pupil, thus getting it out of danger. Hot water applied to the closed eyelids is also a valuable agent in these affections. The water may be applied by means of cloths or sponges. When there is any constitutional cause tonics must be given. When pus is very marked in the anterior chamber it may be evacuated by means of an operation called paracentesis cornea. That is merely tapping the chamber by a narrow knife at the corneal margin, the same operation that is performed for the removal of filaria from the eye. The foregoing methods of treatment will, in most cases, cure inflammation of the cornea. For the relief of the opacities, obstructing vision, after these inflammations, surgical measures have to be resorted to. When the opacity covers the pupil, or where the iris is entangled in the cicatrix an artificial pupil can be made by performing an iridectomy; that is, removing a piece of the iris at a point opposite clear cornea. Through this new opening the animal will see. The white spot can be made less prominent by tatooing with India ink in the same manner that the skin is stained. Of course such eyes are never in as perfect condition as before the disease began. Deep scars of the cornea cannot be removed by medication, although pounded glass, formerly, was much used for this purpose. The superficial scars are sometimes made less prominent, it is thought by some, by the use of calomel dusted into the eye frequently, or by the use of the yellow oxide of mercury mixed with vaseline, the proportion of five grains to the half ounce, this is put into the eye. For myself I think medicines do very little, and that time causes their disappearance more than anything else. Early treatment of corneal inflammations will largely decrease these scars so disastrous to good vision.

*Obstruction of the Tear Ducts.*—The condition spoken of as "watery eye" is one often of great annoyance to the animal, and also impairs the usefulness of the eye. It is due to an inflammation of the mucous membrane lining the lachrymal canal, which membrane is continuous with that of the nose and throat. Catahrral inflammations of the nose and pharynx cause the majority of the cases of lachrymal obstruction by direct continuity of inflammation. The obstruction may be due to either swelling of the lining membrane or to a narrowing of the canal by bony pressure.

When produced by either, the symptoms are the same. A constant flowing of tears over the eyelid on to the nose and face, the conjunctiva is always red and inflamed by the constant presence of the acid tears. By pressing in at the inner angle of the eyelid, right over the head of the lachrymal sac, tears and, in some instances, muco-pus can be squeezed out. Sometimes an abscess forms in the lachrymal sac, giving rise to much pain and considerable swelling, which may either open through the natural passages or open on the face, forming a fistula.

We frequently see cases of ulcer of the cornea that are directly caused by obstruction of the tear ducts, the irritating tears acting as a poison to the eye. It thus behooves us to examine carefully the cause, in order that we may scientifically treat all our patients.

Treatment is mechanical and consists in removing the obstruction. After having satisfied ourselves of the disease it will be necessary to open, with a narrow knife with a probe point, the little canal that is near the edge of the lower eyelid. The probe point is introduced into the opening of the canal (which can be easily seen) and the knife passed to the lachrymal sac, thus cutting off the top of the canal, in other words converting the canal into an open trough. This opening made, a fine probe, like a filiform urethral bougie, may be introduced through the entire length of the nasal duct. Should the stricture be so dense that a small bougie will not pass, a knife may be passed down and the stricture incised, after which the probe may be passed. This procedure may be considered by some impracticable, but it is not, for as long ago as 1850 Percival did this operation with success. The use of eye lotions do very little good, as the obstruction still remains, and nothing but a surgical procedure will rectify it. After the operation is performed the parts must be kept clean and the canal open for the first few days by the use of a probe or bougie. The nasal duct may also be syringed out by astringent solutions, thus medicating the entire lining of the tube. Solutions of powdered alum, one drachm to the pint of water, or weak solutions of nitrate of silver, two to five grains to the ounce, may be used with great benefit; the nozzle of the syringe being introduced into the slit up canal on the edge of the lid.

*(To be continued.)*

